# Oral findings in paediatric patients with severe heart, liver, and kidney failure prior to organ transplantation

**DOI:** 10.1007/s40368-024-00879-6

**Published:** 2024-03-14

**Authors:** I. Landén, A. E. Olander, E. Salmela, T. Jahnukainen, H. Ruokonen, H. Alapulli, J. Helenius-Hietala

**Affiliations:** 1 Department of Oral and Maxillofacial Diseases, Helsinki University Hospital and University of Helsinki, P.O. Box 281, 00029 HUS Helsinki, Finland; 2 Department of Oral and Maxillofacial Diseases, New Children’s Hospital, Helsinki University Hospital and University of Helsinki, Helsinki, Finland; 3 Department of Pediatric Nephrology and Transplantation, New Children’s Hospital, Helsinki University Hospital and University of Helsinki, Helsinki, Finland

**Keywords:** Dental caries, Teeth, Developmental defects of enamel, Oral hygiene, Children

## Abstract

**Purpose:**

Organ transplantation is an effective treatment for children with severe heart, liver, and kidney diseases. These patient groups may have more oral and dental diseases than healthy controls. It is important to eliminate oral infection foci before transplantation and to maintain good oral health to avoid potential post-transplant complications. The aim of this study was to describe and compare oral health in Finnish paediatric heart, liver, and kidney transplant recipients prior to organ transplantation.

**Methods:**

Eighty-six children who received a heart (*n* = 21), liver (*n* = 19), or kidney (*n* = 46) transplant in Finland during the years 2014–2018 were included in this study. The inclusion criterion was a pre-transplantation oral examination. Oral hygiene, enamel anomalies, and the number of decayed, missing, and filled teeth (dmft/DMFT) were analyzed retrospectively from medical and dental records and compared between the three patient groups.

**Results:**

Children with liver (*p* = 0.043) or heart (*p* = 0.047) disease had higher combined primary and permanent dentition dmft/DMFT scores compared to children with kidney disease. A higher combined dmft/DMFT score was associated with poor oral hygiene (*p* = 0.005). No significant differences in oral hygiene between the patient groups were found. Furthermore, all patient groups had a high prevalence of developmental dental defects.

**Conclusion:**

Children with liver or heart disease seem to have a higher combined dmft/DMFT score, indicating a higher prevalence of caries compared to children with kidney disease. Prevention of dental caries, along with promoting a good oral hygiene routine and regular check-ups, is suggested in these patient groups.

## Introduction

Organ transplantation is an effective treatment for paediatric patients with severe heart, liver, and kidney conditions. Congenital defects, such as congenital heart defects (CHD), biliary atresia (BA), and anomalies of the kidney and urinary tract (CAKUT), constitute the most common causes for organ transplantation (Jahnukainen et al. 2016; Raissadati et al. 2016; de Ville de Goyet et al. 2022). After transplantation, immunosuppressants become a necessity. Life-long surveillance is needed to detect and diagnose potential problems, such as infections and tumours (Malik et al. 2015).

The current literature regarding oral health in children with severe chronic organ failure is controversial. Paediatric patients with severe chronic heart, liver, and kidney diseases may have issues with their oral health due to their primary condition or medication. On the one hand, studies have reported green tooth colouration in both primary and permanent dentition, along with higher decayed, missing, filled teeth (DMFT) scores, higher plaque indexes, and more developmental defects of the enamel (DDE) in paediatric patients with chronic liver disease compared to healthy controls (Amaral et al. 2008; Sandoval et al. 2017; Alanzi et al. 2019). On the other hand, Sheehy et al. (2000) showed no difference in DMFT scores between these children and healthy controls. In children with chronic kidney disease, studies have reported elevated levels of calculus, enamel hypoplasia, dry mouth, and poor oral hygiene (Gupta et al. 2015), but less dental caries compared to healthy controls (Gupta et al. 2015; Andrade et al. 2015). According to some studies, children with CHD may exhibit changes in the structure of enamel and dentin (El-Hawary et al. 2014). Findings concerning dental caries in children with CHD are controversial. Some studies show no difference in caries prevalence between these patients and healthy controls (Karhumaa et al. 2021), whilst others show a higher caries prevalence among children with CHD (Stecksén-Blicks et al. 2004; Pourmoghaddas et al. 2018). The connection between oral and systemic health is being increasingly investigated, and an association between the two has been suggested (Meurman & Bascones-Martinez 2021).

Studies conducted thus far broadly depict oral health characteristics in paediatric patients with chronic heart, liver, and kidney diseases (Sheehy et al. 2000; Lucas & Roberts 2005; Gupta et al. 2015; Olczak-Kowalczyk et al. 2014; Hughes et al. 2019). However, information on the oral health of paediatric organ transplant recipients prior to transplantation remains scarce and such studies have, to the authors’ knowledge, never before been conducted in paediatric patients in Finland. All transplant candidates in Finland undergo oral examination as a part of the pre-transplant evaluation, if the patient’s condition allows it.

The aim of this study is to describe oral health in paediatric patients prior to organ transplantation and compare findings between heart, liver, and kidney patients. We hypothesize differences between the groups in their oral health status due to the different underlying systemic diseases and medication.

## Material and methods

This retrospective study was approved by the administration of Hospital District of Helsinki and Uusimaa (HUS/26/2018, 13.12.2018) and was performed in accordance with the Declaration of Helsinki. The data were retrospectively collected from medical records by one of the authors (IL).

**Fig. 1:**
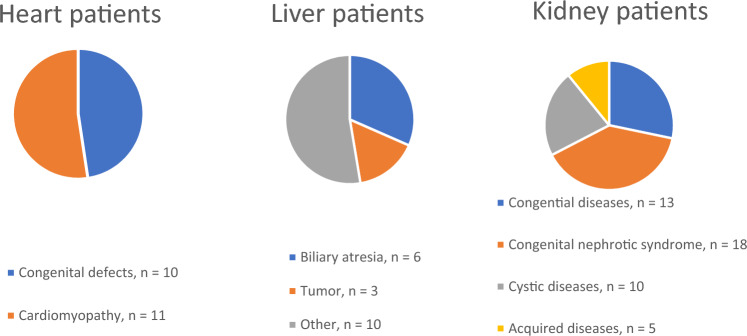
Pie charts showing the distribution of indications for organ transplantation in the heart, liver, and kidney patient groups

This study included 86 paediatric patients, with a median age of 7.5 years (range 0.6–17.6 years) who had received a heart (*n* = 21), liver (*n* = 19), or kidney (*n* = 46) transplant during the years 2014–2018 at the Children’s Hospital, Helsinki University Hospital, Finland, which is the only centre for organ transplantations in Finland. All pre-transplant evaluations were performed via the same protocol. Patients lacking a dental examination prior to transplantation, mainly due to some form of acute organ failure, were excluded from this study. The most common underlying indications for organ transplantation were cardiomyopathy and CHD in patients with heart transplants, BA in patients with liver transplants, and congenital nephrotic syndrome of the Finnish type in patients with kidney transplants. The underlying diseases are presented in more detail in Fig. 1.

All patients included in this study had undergone an oral examination prior to transplantation. This oral examination was performed by a resident or specialist in paediatric dentistry. The oral examination consisted of careful examination of the oral mucosa and dentition. Dental inspection mirror, probe and fibre-optic transillumination were used. X-rays (bitewing and panoramic tomography) were taken when needed. No radiological data is analysed in this study. The dentition stage, possible DDE (hypomineralization and hypoplasia), and discolouration were registered, and information on oral hygiene and possible vomiting was collected. For this study, oral hygiene was evaluated subjectively by a dentist and was defined as being either good or poor. Good oral hygiene was defined as having no or only a small amount of plaque on a few teeth, and poor oral hygiene was defined as having an abundance of plaque on nearly all teeth. For each patient, a DMFT score was registered for both permanent (DMFT) and primary (dmft) dentition. A combined dmft/DMFT score including scores for both permanent and primary dentition in each patient group was used in the analyses due to the limited sample size.

### Statistical analysis

Data analysis was performed using IBM SPSS Statistics 27. Data are given as count (%) or mean ± SD. Continuous variables were assessed for normality using histograms and a Shapiro–Wilks test. None of the continuous variables were normally distributed. Differences between groups were assessed using a Fisher–Freeman–Halton exact test, a Mann–Whitney U test, and a Kruskal–Wallis test, with a *Z*-test for proportion and Dunn–Bonferroni post hoc tests used as necessary. Post hoc tests were Bonferroni corrected. Two-tailed *p*-values of < 0.05 were considered significant.

## Results

Background characteristics of children awaiting heart, liver or kidney transplantations are shown in Table 1. Neither median age at transplantation nor sex differed significantly between the three groups. The kidney transplant group had a statistically significantly higher percentage of patients with primary dentition compared to the heart transplant group (*p* = 0.009). No other statistically significant differences in the dentition stages between the groups were found.

**Table 1:** Background characteristics of children with heart, liver, and kidney transplantations

Parameter	Heart	Liver	Kidney	p-value
No. of patients	21	19	46	
Age at transplantation (years)	10.50 (9.91)	7.92 (8.83)	2.46 (11.68)	0.098
Sex (male/female)				0.599
Male	9 (42.9%)	8 (42.1%)	25 (54.3%)	
Female	12 (57.1%)	11 (57.9%)	21 (45.7%)	
Dentition period				0.012*
Not erupted	1 (4.8%)	1 (5.3%)	0 (0.0%)	
Primary	5 (23.8%)	7 (36.8%)	29 (63.0%)	
Mixed	10 (47.6%)	10 (52.6%)	12 (26.1%)	
Permanent	5 (23.8%)	1 (5.3%)	5 (10.9%)	

Parameters are given as *n* (%), except for age, which is given as the median (IQR)

*p*-values correspond to the Fisher–Freeman–Halton exact test, z-proportions test (Bonferroni corrected). *p*-values for continuous variables correspond to the Kruskal–Wallis test

*The proportion of patients with primary dentition was significantly higher in the kidney group compared to the heart group, *p*-value = 0.009

Regarding oral hygiene, no statistically significant differences were found between the groups in either primary, mixed, or permanent dentition, or when all dentitions were analysed together (Table 2). Nonetheless, patients with poor oral hygiene had significantly higher combined dmft/DMFT scores compared to patients with good oral hygiene (poor oral hygiene (3.5 ± 3.2) vs good oral hygiene (0.7 ± 1.9), *p* = 0.032, data not shown in table).

**Table 2:** Oral hygiene (good/poor) in children before heart, liver, and kidney transplantations

Dentition	Heart	Liver	Kidney	*p*-value
Primary	4 (100.0%)/0 (0.0%)	7 (100.0%)/0 (0%)	19 (95.0%)/1 (5.0%)	1.000
Mixed	9 (100.0%)/0 (0%)	7 (77.8%)/2 (22.2%)	10 (83.3%)/2 (16.7%)	0.521
Permanent	4 (80.0%)/1 (20.0%)	1 (100.0%)/0(0.0%)	5 (100.0%)/0(0.0%)	1.000
All dentitions together	17 (94.4%)/1 (5.6%)	15 (88.2%)/2 (11.8%)	34 (91.9%)/3 (8.1%)	0.741

Oral hygiene (good/poor) is given for each group as n (%). *p*-values correspond to the Fisher–Freeman–Halton exact test. Data were missing on 3 children in the heart transplant group, 2 children in the liver transplant group, and 9 children in the kidney transplant group

**Fig. 2:**
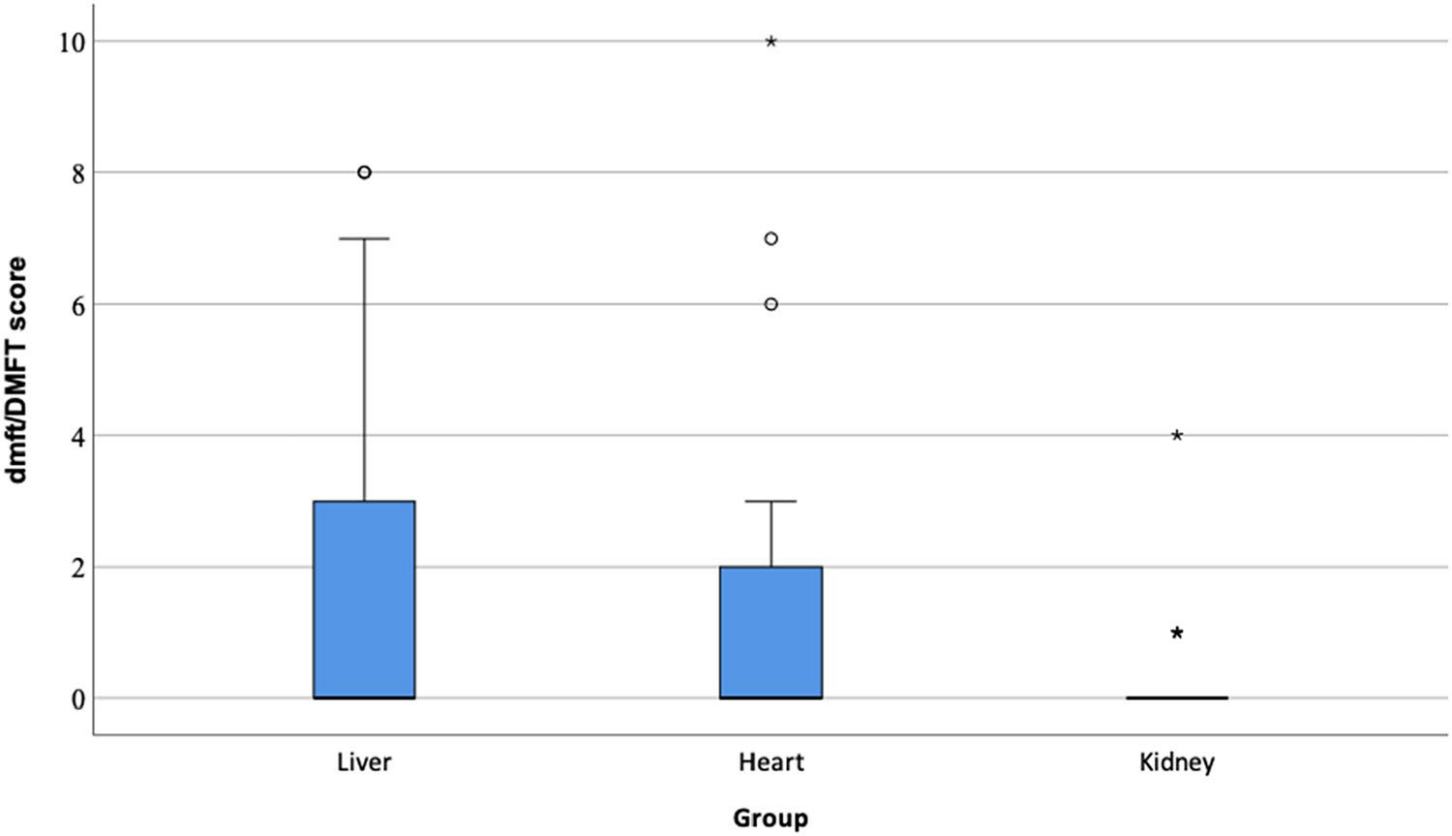
The distribution of combined dmft/DMFT scores in children prior to liver, heart, and kidney transplantation. The x-axis shows the different groups. The y-axis shows the dmft/DMFT score. There was a significant difference between the groups in the Kruskal¬–Wallis test (*p* = 0.010). The liver group had a significantly higher dmft/DMFT score compared to the kidney group (*p* = 0.043, Dunn–Bonferroni test) and the heart group had a significantly higher dmft/DMFT score compared to the kidney group (*p* = 0.047, Dunn–Bonferroni test). Whiskers indicate the non-outlier maximum value, the circles denote mild outliers (1.5 × IQR above third quartile), and an asterisks denote extreme outliers (> 3.0 × IQR above third quartile)

When analyzing combined dmft/DMFT scores between the heart (mean 1.6 ± 2.9), liver (mean 1.8 ± 2.9), and kidney (mean 0.2 ± 0.7) groups, a significant difference was found (*p* = 0.010). In pairwise comparisons, both the liver group (*p* = 0.043) and the heart group (*p* = 0.047) had a significantly higher mean combined dmft/DMFT score compared to the kidney group. The distribution of combined dmft/DMFT scores is described in Fig. 2.

Tooth colouration and DDE (hypomineralization and hypoplasia) in the three groups are described in Table 3. Only one patient with BA had green tooth colouration. Furthermore, some tooth colouration might have been superficial. There was no significant difference in the prevalence of tooth colouration or DDE between the groups. However, DDE prevalence was high in all patient groups (heart 65.0%, liver 27.8%, kidney 41.3%).

**Table 3:** Developmental defects of enamel (DDE) and tooth colouring in children prior to heart, liver, and kidney transplantations

Dental anomaly	Heart	Liver	Kidney	*p*-value
Colouring	0 (0.0%)	3 (16.7%)	4 (9.3%)	0.247
DDE	13 (65.0%)	5 (27.8%)	19 (41.3%)	0.061

Data given as n (%). *p*-values correspond to the Fisher–Freeman–Halton exact test, z-proportions test (Bonferroni corrected). 8 patients were missing data on colouring, 2 patients were missing data on DDE

*DDE* developmental defects of enamel

Gingival overgrowth was present in two kidney patients before organ transplantation. Both were taking calcium channel blockers. Both had good oral hygiene. There were also 11 other kidney patients with calcium channel blockers who did not have gingival overgrowth. No gingival overgrowth was found in liver or heart patients before organ transplantation.

Vomiting and its association with erosion or attrition was also analysed. Kidney patients were more prone to vomiting compared to heart patients (*p* = 0.023). When comparing patients with and without erosion/attrition we found no difference in the proportion of patients who experienced vomiting. The results are shown in Online Resource 1.

## Discussion

The main finding in our study was that the combined dmft/DMFT scores in children with liver disease and with heart disease were significantly higher than in children with kidney disease. The highest combined dmft/DMFT scores were found in children with liver disease, indicating a higher caries prevalence compared to children with heart or kidney disease. Although poor oral hygiene associated with a higher dmft/DMFT score, no significant difference in oral hygiene was found between the groups.

Studies on caries prevalence in paediatric patients with heart, liver, and kidney disease are inconclusive. One study shows that children with chronic liver disease have a higher caries prevalence compared to healthy controls (Alanzi et al. 2019). These findings align with the results of our study. Furthermore, Shiboski et al. (2009) reported high caries prevalence in children with liver transplants. A low caries prevalence has been reported in children with kidney disease (Nunn et al. 2000; Al-Nowaiser et al. 2003; Shiboski et al. 2009; Gupta et al. 2015) and in a meta-analysis of individuals with chronic kidney disease (Limeira et al. 2019). Furthermore, children with CHD have been reported by some Nordic studies to have poorer oral health and more caries than healthy controls (Stecksén-Blicks et al. 2004; Sivertsen et al. 2016). Similar results have been shown in a systematic review article (Karikoski et al. 2021). Similarly, a Finnish study showed no significant difference in caries prevalence between children with CHD and healthy controls (Karhumaa et al. 2021). In our study, children with heart disease had significantly higher combined dmft/DMFT scores compared to children with kidney disease. In a register study of Finnish children in 2003, 5-year-olds recorded an average combined dmft/DMFT score of 0.9, 12-year-olds a score of 1.2, and 17-year-olds a score of 4.0 (Suominen-Taipale and Widström, 2009). Compared to these findings, our study shows higher dmft/DMFT scores, indicating a higher caries prevalence in children with either heart (mean combined dmft/DMFT 1.6, median age 10.5) or liver disease (mean combined dmft/DMFT 1.8, median age 7.9) awaiting organ transplantation.

Previous studies report that children with kidney disease have more calculus build-up compared to healthy controls (Davidovich et al. 2009). The saliva is more alkaline secondary to chronic kidney disease, decreasing the caries risk, but also increasing the accumulation of calculus (Davidovich et al. 2009; Subramaniam et al. 2012). This could explain why in our study, children with kidney disease had combined dmft/DMFT scores close to zero (mean combined dmft/DMFT 0.2) and significantly lower scores than children with liver or heart disease. Furthermore, kidney patients were younger on average than patients in the other groups, which might be a contributing factor to their lower caries prevalence. We found that patients in the kidney group experienced significantly more vomiting compared to patients in the heart group. Vomiting did not, however, seem to associate with erosion/attrition of the teeth, which may partly be explained by the young age at which they received a transplantation.

In our study, we did not see a significant pre-transplant difference in oral hygiene between the three organ transplant groups. In general, we found that oral health was good among these patients. Heart and liver patients showed high combined dmft/DMFT scores and, therefore, attention should be given to these patients, especially regarding preventive measures. Previous studies show an increased amount of gingival overgrowth in children after organ transplant due to immunosuppressive medication (Wondimu et al. 2001), especially if calcium blocking hypotensive drugs are used in combination with cyclosporin A (Bökenkamp et al. 1994; Lucas & Roberts, 2005). Furthermore, a higher prevalence of dental plaque is associated with drug-induced gingival overgrowth, which emphasizes the importance of good oral hygiene (Lin and Yang 2010). In our study, only two patients with calcium channel blockers had gingival overgrowth before transplantation. Both had good oral hygiene. Maintaining good oral health is especially important in all three patient groups, since they are prone to infectious complications due to their primary condition and to the life-long immunosuppressive medication they require post-transplantation (Nappalli & Lingappa 2015; Campbell et al. 2020; Kwak et al. 2020)*.*

Children with CHD have been reported to exhibit changes in enamel and dentin structure (El-Hawary et al. 2014) and show more enamel hypoplasia compared to healthy controls (Hallet et al. 1992). DDE has also been reported in children with chronic kidney disease and with chronic liver disease (Gupta et al. 2015; Alanzi et al. 2019). The proportion of DDE between the three patient groups did not significantly differ. However, many patients did have DDE. Both liver and kidney transplant recipients are shown to have more dental and bone abnormalities compared to the general population. One contributing factor is disturbances in the calcium and phosphate balance and metabolism of these patients. This can occur due to conditions that are present before organ transplantation, but also due to post-transplant immunosuppression (Olczak-Kowalczyk et al. 2012). Furthermore, malnutrition, haemodynamic alterations, infective endocarditis, medication, and hypoxia have been suggested as factors in the formation of enamel anomalies in heart patients (El-Hawary et al. 2014). Developmental defects of the enamel are shown to increase the risk of dental caries, both in primary and permanent dentition (Costa et al. 2017; Vargas-Ferreira et al. 2015).

Case reports also show green tooth colouration in children with BA (Amaral et al. 2008; Rakauskaite et al. 2014). In our study, there was no significant difference in tooth colouration between the groups. Three liver patients (16.7%) and four kidney patients (9.3%) had some form of tooth colouration of which some might have been superficial in some patients. One of the liver patients with BA had green tooth colouration in two teeth. No discolouration was observed in any heart patient. Use of iron solutions is a known risk factor for tooth discolouration. Cholestatic liver disease, especially BA, can cause green tooth colouration during the formation of the teeth (Vidigal et al. 2020). At our centre, clearance of jaundice is achieved in the vast majority (75%) of BA patients after Kasai portoenterostomy, which may explain why relatively few liver patients had coloured teeth. On the other hand, due to the high incidence of congenital nephrosis of the Finnish type, many kidney transplant patients in our study were infants and were on oral iron solution.

This study is particularly important as it is the first study to describe oral health in children undergoing pre-transplantation evaluations in Finland. To the best of the authors’ knowledge, this is also the first study to compare oral health between paediatric patients receiving heart, liver, and kidney transplants. One limitation of this study is that it lacks a healthy control group. However, including a healthy control group was not possible in this study setup. Further limitations are the difference in group sizes and the small sample size. Unequal group sizes might affect the statistic power. To perform adequate analysis, we were constrained to use quite heterogeneous groups concerning age and dentition stage and were not able to group the patients into more specific subgroups. These are possible confounding factors especially concerning oral hygiene routines affecting caries and other oral diseases.

However, this study is highly inclusive, as it comprised all children in Finland undergoing pre-transplant evaluations for heart, liver, and kidney transplantations with sufficient dental data during the years 2014–2018; this was possible as all organ transplantations are centralised to HUS.

## Conclusions

In conclusion, children with liver and heart disease seem to have a higher prevalence of decayed, missing, and filled teeth compared to children with kidney disease although their oral hygiene was mainly good. Furthermore, children with severe heart, liver, or kidney disease seem to have a high prevalence of DDE. Attention should be paid to the prevention of dental caries in these patient groups. Regular dental examinations, preventive care, and promoting a good oral hygiene routine in these patient groups from an early age is key in preventing oral diseases.
